# Research on the Rutting Resistance of Asphalt Improved by Nitrogen-Rich Soybean Biochar

**DOI:** 10.3390/ma19173639

**Published:** 2026-08-27

**Authors:** Cuicui Sun, Zhe Li, Junxia Yang, Xuanchen Zhou, Yanling Wu, Haocheng Zhang, Changhao Si, Xiaofeng Tian, Chiara Riccardi, Dedong Guo

**Affiliations:** 1 Shandong Key Laboratory of Technologies and Systems for Intelligent Construction Equipment, School of Civil Engineering, Shandong Jiaotong University, Jinan 250300, China; 204150@sdjtu.edu.cn (C.S.); 25307016@stu.sdjtu.edu.cn (Z.L.); 230764220@stu.sdjtu.edu.cn (X.Z.); 230764309@stu.sdjtu.edu.cn (H.Z.); 230764313@stu.sdjtu.edu.cn (C.S.); 401002@sdjtu.edu.cn (D.G.); 2 Shandong Science and Technology Service Development and Promotion Center, Jinan 250300, China; 3 Department of Civil and Industrial Engineering, University of Pisa, Largo Lucio Lazzarino 1, 56126 Pisa, Italy; chiara.riccardi@unipi.it

**Keywords:** nitrogen-rich biochar, surface functionalization, asphalt binder, rheological properties, aging resistance

## Abstract

Asphalt pavements suffer from progressive deterioration during service life due to aging, while conventional polymer modifiers raise environmental concerns regarding recyclability and volatile organic compound emissions. Functionalized-biochar derived from renewable biomass offers a sustainable alternative through its tunable surface chemistry and porous structure. This study develops and evaluates a nitrogen-rich, surface-functionalized biochar as a multifunctional asphalt binder modifier and elucidates the synergistic roles of inherent nitrogen and post-synthetic functionalization in governing binder performance. To this end, soybean powder was pyrolyzed to prepare the biochar precursor, which was subsequently characterized to determine its suitable pyrolysis temperature and surface properties. These analyses identified 300 °C as the preferable pyrolysis temperature within the tested range of 200–500 °C, maximizing biochar yield and achieving favorable surface physicochemical properties including the iodine adsorption value, oil absorption value, surface functional groups and pore morphology. To improve surface functionality, the biochar underwent a two-step modification: nitric acid oxidation followed by hydroxymethylation, hereinafter referred to as functionalized-biochar. The influence of functionalized-biochar content on asphalt binder performance was evaluated across dosages of 10–20 wt%, and 15 wt% was identified as the recommended content. Asphalt binder modified with 15 wt% functionalized-biochar exhibited improved high-temperature performance, as evidenced by enhanced rutting resistance factor G*/sin δ values obtained from dynamic shear rheometer testing. Aging resistance also improved, reflected in a lower complex modulus aging index and a higher phase angle aging index. At 15 wt% dosage, the softening point increased by 6 °C, and ductility rose by 10.6 cm relative to the base asphalt (1.9 cm), corresponding to a 557.9% improvement. Penetration declined by 17%, while the complex modulus aging index decreased from 3.44 to 1.68. Notably, the experimental program in this study was limited to binder-scale characterization.

## 1. Introduction

Highways serve as the backbone of the global logistics network, facilitating economic growth through their unmatched flexibility and efficiency [1]. Asphalt concrete, owing to its superior rheological properties, ride comfort, and cost-effective maintenance, is widely adopted for high-grade pavement construction [2,3]. However, the synergistic degradation caused by heavy traffic loads and extreme climatic conditions often leads to premature pavement failures—such as rutting, fatigue cracking, and moisture damage—thereby curtailing the intended design service life [4]. Conventional asphalt mix design concentrates on adjusting aggregate gradation and asphalt content to satisfy basic road performance requirements. Such design is limited, as it cannot fundamentally improve the inherent properties of asphalt binders and fails to adequately address aging, high-temperature rutting, and low-temperature deterioration under variable service conditions. Since conventional mix design only optimizes mixture composition rather than the binder matrix, modifying asphalt binders has emerged as the most viable strategy for enhancing pavement durability. Beyond optimizing binder performance, transitioning road construction toward circular economy models has become an essential pathway to reduce resource consumption and construction waste [5]. Agricultural residues such as soybean powder can be converted into carbonaceous biochar, serving as a modifier for asphalt binders. This upcycling of agricultural biomass simultaneously mitigates waste disposal pressure and improves long-term pavement durability, aligning with sustainable infrastructure goals.

Biochar, derived from carbonaceous biomass, has attracted substantial attention as a sustainable asphalt modifier [6,7,8]. It effectively enhances the binder’s high-temperature stiffness and viscosity while addressing the environmental challenges associated with conventional polymer modifiers [9,10]. Beyond simple mechanical reinforcement, biochar’s porous structure and complex surface chemistry facilitate the immobilization of hazardous gases [11,12,13,14,15] and provide a physical barrier that retards photo-oxidative and thermo-oxidative aging [16,17,18,19]. Nevertheless, the performance of biochar-modified asphalt is heavily contingent upon the physicochemical properties of the biochar, which are governed by biomass feedstock selection and thermochemical processing conditions [20,21,22].

Recently, nitrogen-enriched biochar has emerged as a promising modifier due to its enhanced interfacial compatibility and adsorption capacity. Studies have demonstrated that externally doped nitrogen (e.g., via chemical impregnation or ammonia treatment) can introduce active functional groups that improve biochar–asphalt interactions [23,24,25,26,27,28]. Parallel efforts have focused on chemical surface modification of biochar to reduce particle re-aggregation and increase active site density [29,30,31]. However, these two strategic pathways—nitrogen enrichment and surface functionalization—have largely been pursued independently. To date, a systematic investigation into the synergistic effects of inherent nitrogen species within the biomass and targeted post-synthetic surface functionalization on asphalt performance remains conspicuously absent. In particular, the mechanism by which surface-modified, nitrogen-rich biochar dictates the dispersion stability and long-term rheological properties of asphalt binders warrants further exploration.

Therefore, this study introduces a distinct strategy that uniquely integrates both attributes by employing naturally nitrogen-rich biomass as a precursor and subjecting it to a mild, two-step surface functionalization. Soybean powder was chosen as a model precursor for this purpose. It possesses high protein and lignin content, which inherently facilitates the formation of abundant oxygen- and nitrogen-containing groups during pyrolysis, a feature verified to enhance adsorption, interfacial compatibility, and dispersion stability [32,33]. Critically, soybean powder is adopted strictly as a research model to elucidate the fundamental role of inherent nitrogen in biochar–asphalt interactions, not as a recommended food-based feedstock. Its high protein content enables the natural formation of pyrrolic-N, pyridinic-N, and graphitic-N during mild-temperature pyrolysis, providing a reproducible platform without costly chemical doping [34,35]. More importantly, the findings are readily extendable to sustainable, non-food, nitrogen-rich waste, such as soybean meal, okara, rapeseed meal, or microalgae, which are abundant by-products of the food and biofuel industries. Their utilization reduces disposal costs, consumes less energy, and aligns with circular economy principles, reinforcing the environmental and economic viability of biochar-modified asphalt systems.

To overcome the inherent limitations of biochar, such as particle reaggregation and weak interfacial adhesion [36], this study specifically adopts a two-step HNO_3_ oxidation + hydroxymethylation functionalization. This mild and controllable method was selected over harsher treatments because it effectively grafts abundant oxygen-containing groups onto the biochar surface without damaging the carbon framework [37]. This process not only reduces particle reaggregation and enhances surface hydrophilicity but also strengthens interfacial interactions with asphalt components [32,38,39]. Importantly, these functional groups act synergistically with the inherent nitrogen species to create a multifunctional protective layer, contributing to UV shielding and antioxidant activities [40]. In summary, while previous studies have explored nitrogen-doped or chemically modified biochar separately, this work presents a comprehensive investigation into the synergistic effects of “inherent nitrogen content” and “post-synthetic surface functionalization.” By systematically evaluating the impact of this functionalized, nitrogen-rich biochar on the mechanical, rheological, and anti-aging properties of asphalt, this study aims to establish a robust performance-mechanism framework that supports the deployment of sustainable, bio-based additives in road infrastructure.

## 2. Materials and Methods

### 2.1. Experimental Program

**Figure 1:**
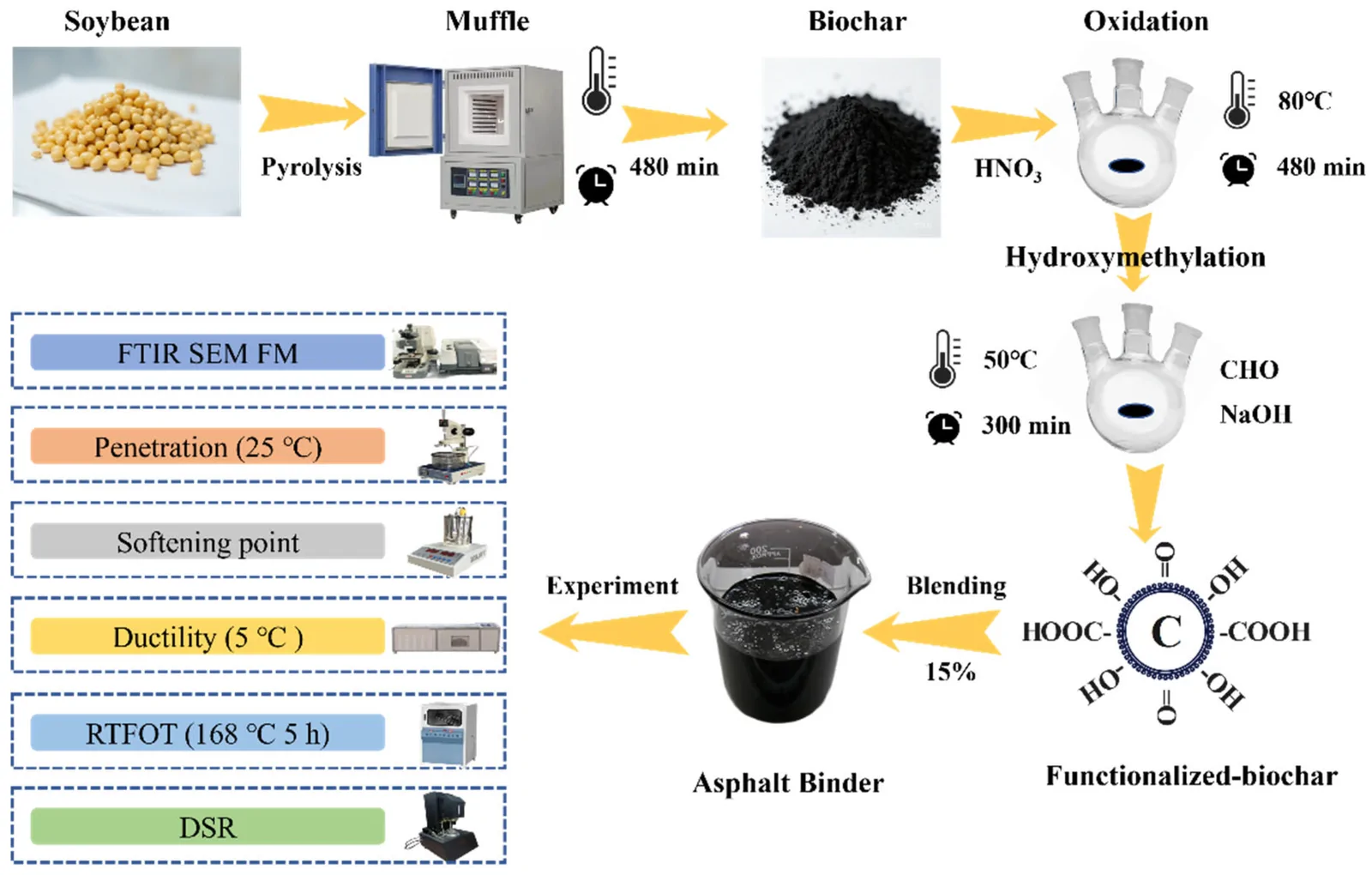
Research framework of this study.

The experimental workflow is presented in Figure 1. Soybean powder was pyrolyzed to obtain biochar, followed by oxidation and hydroxymethylation treatments to synthesize functionalized-biochar. After blending the functionalized-biochar (15 wt%) with the base asphalt, a series of tests were carried out. Fourier transform infrared spectroscopy (FTIR) (Tensor27, Bruker GmbH, Ettlingen, Germany), scanning electron microscopy (SEM) (Sigma 500, Carl Zeiss Microscopy Ltd., Cambridge, UK), and fluorescence microscopy (FM) (AXioscope 5, Carl Zeiss Microscopy Ltd., Cambridge, UK), were used for microstructural observation. Penetration, softening point, and ductility tests characterized the conventional physical properties. Furthermore, the rolling thin film oven test (RTFOT) (SYD-0610, Shanghai Changji Geological Instrument Factory, Shanghai, China) aging procedure and dynamic shear rheometer (DSR), (BOHLIN GEMINI II, Malvern Panalytical Ltd., Malvern, Worcestershire, UK) tests were conducted to assess the anti-aging capacity and rheological behavior of the asphalt binders.

### 2.2. Base Asphalt

In this study, 70# base asphalt supplied by Heze Zhancheng Testing Technology Co., Ltd. (Heze, China) was used as the base asphalt. Table 1 summarizes the basic properties of the selected base asphalt, and the evaluation was conducted in accordance with the established standard, JTG 3410-2025 [41].

### 2.3. Preparation and Physical Performance of Biochar

**Figure 2:**
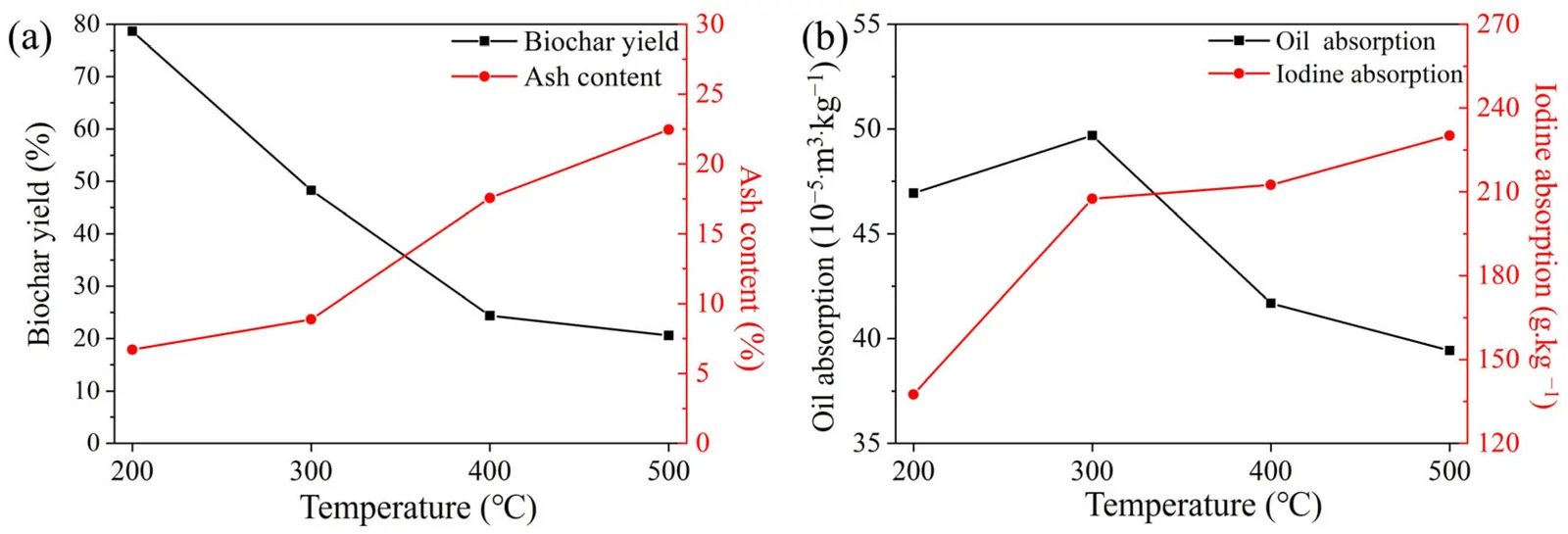
(**a**) Biochar yield and ash content, (**b**) oil and iodine absorption values of biochar.

The soybean powder was placed in a clean crucible and then transferred to a furnace under a continuous flow of high-purity nitrogen (N_2_) to provide an inert atmosphere. After calibrating the furnace, its temperature was set at 200 °C and held for 8 h. After pyrolysis, the crucible was allowed to cool to room temperature, and the resulting biochar was ground repeatedly until no visible particles remained. The biochar yield was defined as the ratio of the mass of the biochar product to the mass of the original soybean powder. Several factors influence biochar yield, including the soybean variety, moisture content, and pyrolysis temperature. When the soybean variety and moisture content are kept constant, pyrolysis temperature becomes the most important factor affecting the yield. The biochar yields at different pyrolysis temperatures were determined by weighing, and the results are shown in Figure 2a. It is clear that the biochar yield is inversely proportional to the pyrolysis temperature: as the temperature increases from 200 °C to 500 °C, the yield decreases from 78.7% to 20.6%. At lower temperatures, the soybean powder absorbs heat, causing the internal moisture to evaporate, transitioning from wet to dry, while its chemical composition remains largely unchanged. As pyrolysis progresses and the temperature rises, macromolecules break down into smaller molecules through complex reactions, such as free-radical reactions, dehydration, decarboxylation, bond cleavage, and molecular rearrangement. The organic matter gradually volatilizes, and thermal decomposition begins. With the separation of volatile components, the remaining solid product is biochar. Water evaporation is a necessary stage in the carbonization process, but the thermal decomposition of organic substances is mainly governed by the pyrolysis temperature.

Ash content refers to the residual inorganic matter remaining after biochar combustion, primarily comprising oxides and inorganic salts of mineral elements. Ash content is mainly influenced by the soybean variety and pyrolysis temperature. Under identical raw material conditions, pyrolysis temperature is the principal factor. The ash content results are presented in Figure 2a. As the pyrolysis temperature increases, the ash content of biochar progressively increases from 6.7% to 22.5%, attributed to the gradual release and removal of volatile organic components, accompanied by the retention and concentration of inorganic residues. Ash content affects the physicochemical properties of biochar and thus its potential applications.

The DBP oil absorption value (denoted as D) measures the minimum volume of dibutyl phthalate (DBP) required to penetrate and adsorb onto the pores and surface of carbonaceous particles. It directly reflects the three-dimensional network characteristics of biochar. The characteristic chain-like aggregates of carbonaceous particles, which form complex fibrous structures through van der Waals interactions, exhibit heterogeneous pore distributions when compacted. This structural feature aligns well with the capillary forces necessary for DBP penetration, enabling sensitive differentiation of pore properties across carbonaceous particles with varying degrees of structural complexity. The D value reflects the biochar’s aggregate structure; a higher D indicates greater oil absorption capacity. As shown in Figure 2b, the D decreases with increasing pyrolysis temperature. As the temperature rises, substantial volatile release causes shrinkage of the carbon framework and collapse of the fibrous aggregate structure, which compacts the particle arrangement and reduces inter-aggregate voids available for DBP adsorption and capillary penetration. Accordingly, the D decreases from 50 × 10^−5^ m^3^·kg^−1^ at 300 °C to 39 × 10^−5^ m^3^·kg^−1^ at 500 °C. At 200 °C, insufficient volatilization of organic matter results in an underdeveloped pore structure and a lower D value compared to that at 300 °C.

The iodine absorption value (I) is positively correlated with the specific surface area of biochar, where a higher value indicates a more developed pore structure. As shown in Figure 2b, the I increased from 137.5 g·kg^−1^ at 200 °C to 230.0 g·kg^−1^ at 500 °C, suggesting an expansion of the specific surface area and enhanced adsorption capacity. Detailed protocols for oil and iodine adsorption measurements are provided in the Supplementary Information. Pore size distribution (Figure S1a in the Supplementary Information) reveals that high-temperature treatment reduces small mesopores and generates larger mesopores, while the peak in the 1–2 nm range indicates minimal micropores. The N_2_ isotherms (Figure S1b) confirm a mesoporous structure: limited N_2_ uptake at low relative pressures further verifies scarce micropores, whereas prominent hysteresis loops reveal abundant macropore formation inside the biochar. In agreement with these findings, SEM images confirm a more defined pore structure for biochar prepared at 300 °C (Figure S2). Elemental analysis (Table S1 in the Supplementary Information) indicates that, with increasing pyrolysis temperature, fixed carbon content increases continuously due to progressive dehydration, deoxygenation, and aromatization that enrich fixed carbon. The nitrogen content peaks at 300 °C, as nitrogen is stabilized in heterocyclic structures, then decreases at higher temperatures due to thermal decomposition. Hydrogen content decreases monotonically with increasing devolatilization, consistent with previous literature [42,43,44]. Consequently, 300 °C is identified as the optimal temperature for biochar production, given the peak nitrogen content at this temperature, which is critical for enhancing surface functionality.

### 2.4. Preparation and Characterization of Functionalized-Biochar

The biochar was modified via HNO_3_ oxidation to introduce active groups, such as OH and –COOH, onto its surface. Subsequently, the oxidized biochar underwent hydroxymethylation using HCHO and NaOH solutions to yield hydroxymethylated functionalized-biochar. In the oxidation process, biochar, dilute HNO_3_, and magnetic stir bars were placed into a 500 mL three-neck flask. The mixture was stirred and refluxed at 80 °C for 8 h. Upon completion, the product was filtered repeatedly with deionized water until the filtrate reached a neutral pH, followed by drying at 50 °C in an oven. The introduction of –OH and –COOH groups increased the polarity of the biochar, thereby enhancing its surface reactivity. For the subsequent hydroxymethylation, the oxidized biochar, NaOH solution, and HCHO were added to a three-neck flask. The reaction was conducted using a constant-temperature magnetic stirrer under a nitrogen atmosphere at 50 °C for 5 h. Finally, the product was washed until neutral to obtain the final functionalized-biochar.

The total acidity (T) and the content of carboxyl and lactone groups (P) of the functionalized-biochar were determined to be 8.5 mol·kg^−1^ and 2.0 mol·kg^−1^, respectively, as detailed in the Supplementary Information. The values were calculated using the following equations:

(1) $$T=\frac{(V_{0}-V)\times C_{\mathrm{HCl}}}{m}$$

where V_0_ and V represent the volumes (mL) of HCl consumed by the biochar and functionalized-biochar, respectively, and C_HCl_ is the concentration of HCl (0.23 mol·L^−1^).

(2) $$P=\frac{(V_{0}^{*}-V^{*})\times C_{\mathrm{NaOH}}}{m}$$

Similarly, V* and V_0_* denote the volumes (mL) of NaOH consumed by the functionalized-biochar and biochar, respectively, with C_NaOH_ being the concentration of NaOH (0.25 mol/L). m is the mass of functionalized-biochar. The Hydroxyl Content (Q) was calculated as Q = T − P, yielding a value of 6.5 mol·kg^−1^. The increase in oxygen-containing functional groups (-OH) confirms the success of the surface modification.

The performance of carbonaceous materials is primarily governed by three factors: the precursor material, the porous structure, and the nature and distribution of incorporated heteroatoms. Among these, oxygen and nitrogen are critical as they influence surface functionality and reactivity [17]. Oxygen-containing functional groups typically introduce acidity and reside at the edges of the carbon skeleton, playing a pivotal role in interfacial chemistry. In contrast, nitrogen-containing groups impart basicity through two mechanisms: (i) the attraction of protons by delocalized π-electrons within aromatic carbon structures, and (ii) the presence of basic surface species capable of proton binding [45]. Consequently, biochar derived from nitrogen-rich biomass often exhibits enhanced adsorption capacity. However, excessive oxygen-containing acidic groups may increase surface hydrophilicity and occupy active adsorption sites, potentially reducing the overall adsorption efficiency.

**Figure 3:**
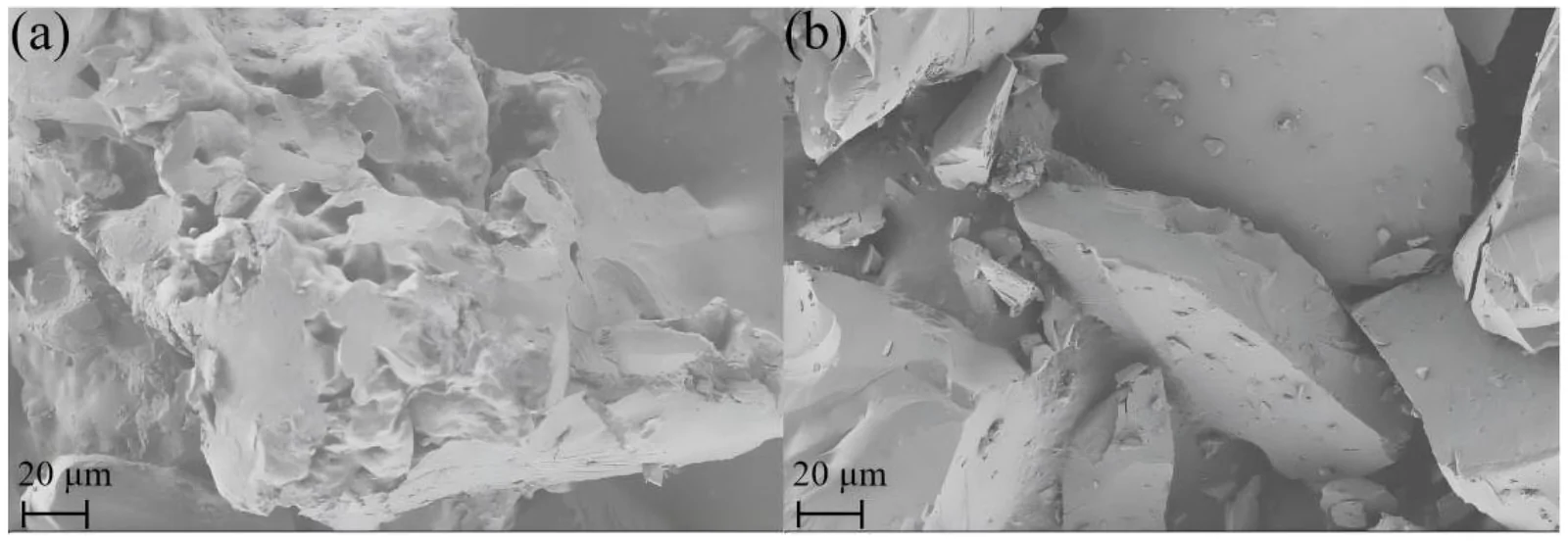
SEM images of (**a**) biochar and (**b**) functionalized-biochar.

SEM images of the biochar and functionalized-biochar are presented in Figure 3a,b. The biochar exhibits a rough surface with irregular morphology and relatively large aggregate particles. Following oxidation and hydroxymethylation, the surface of the functionalized-biochar appears smoother with a notably reduced particle size. The larger size of the biochar is likely attributable to high surface energy and a strong tendency for particle agglomeration, leading to the formation of secondary structures. The two-step modification process effectively disrupts these secondary aggregates, resulting in improved surface continuity and a refined morphology for the functionalized-biochar.

Figure S3 shows the FTIR spectra of the biochar and functionalized-biochar. The biochar exhibits weak characteristic absorption peaks associated with hydroxyl and carbonyl groups. In contrast, the functionalized-biochar displays a prominent peak within the 1730–1670 cm^−1^ range, corresponding to the stretching vibration of the carbonyl group (C=O). This peak is further broadened by the coupling effect of C–N stretching vibration and N–H bending vibration, confirming the presence of nitrogen-containing functional groups—a finding consistent with the elemental analysis. This region is characteristic of ketones, aldehydes, lactones, or carboxyl groups. Additionally, characteristic peaks corresponding to C–O stretching are observed between 1000 and 1300 cm^−1^. The broad absorption peak at approximately 3440 cm^−1^ is assigned to O–H stretching vibration [46]. Furthermore, the strong N–H stretching vibrations of amines in the 3300–3500 cm^−1^ region provides additional evidence of successful nitrogen incorporation. To ensure the accuracy of the FTIR results, all functionalized-biochar samples were rigorously washed with deionized water until the filtrate reached a neutral pH, thereby removing residual reagents. It is noteworthy that the two-step chemical modification not only enhances dispersion but also introduces functional groups that confer anti-aging, adsorption, and interfacial reinforcement properties [39,47]. These multifunctional benefits justify the associated complexity, as evidenced by the improved rheological properties and aging resistance of the modified asphalt.

### 2.5. Preparation of Functionalized-Biochar-Modified Asphalt

The base asphalt was preheated at 135 °C for 3 h. The biochar and functionalized-biochar were then incorporated into the container in multiple stages to ensure uniform distribution. The mixture was subjected to high-shear mixing at 150 °C with an initial shear rate of 3000 rpm. Once all the biochar was added, the shear rate was increased to 4000 rpm for an additional 30 min. Three dosages (10, 15, and 20 wt%) were examined. Given the inherent tendency of biochar particles to agglomerate, this high-shear method was essential to achieve homogeneous dispersion within the base asphalt.

### 2.6. Experimental Methods

The materials were characterized via several techniques. SEM was used to examine the microscopic morphology. FTIR (4000–400 cm^−1^) was employed to compare functional groups in the biochar and asphalt samples before and after short-term aging. FM was used to observe the phase distributions in the asphalt samples. Short-term aging was simulated using the RTFOT following ASTM D2872 [48]. Asphalt samples (approximately 35 g) were placed in cylindrical glass bottles and rotated in an oven at 163 °C for 85 min at 15 rpm.

Basic physical properties were evaluated according to the Chinese specification JTG 3410-2025 [41], including penetration (25 °C, 5 s; T0604-2011), softening point (initial temperature 5 ± 1 °C, 5 °C/min; T0606-2011), and ductility (5 °C, 5 cm/min; T0605-2011). Aging resistance was assessed using mass loss, residual penetration ratio (RPR = P_2_/P_1_ × 100%), and softening point increment (SPI = SP_2_ − SP_1_), where P_1_ and P_2_ are the penetration depths of original and aged asphalt, respectively, and SP_1_ and SP_2_ are the corresponding softening points (in °C). Rheological properties were measured using a DSR test based on the SHRP (Strategic Highway Research Program) guidelines. Testing was conducted with a 25 mm parallel-plate geometry (1 mm gap) over a temperature range of 46–70 °C (6 °C/min) at a frequency of 10 rad/s. Key parameters obtained included the complex modulus (G*), phase angle (δ), and rutting resistance factor (G*/sin δ).

## 3. Results and Discussion

### 3.1. Microstructural Performance

**Figure 4:**
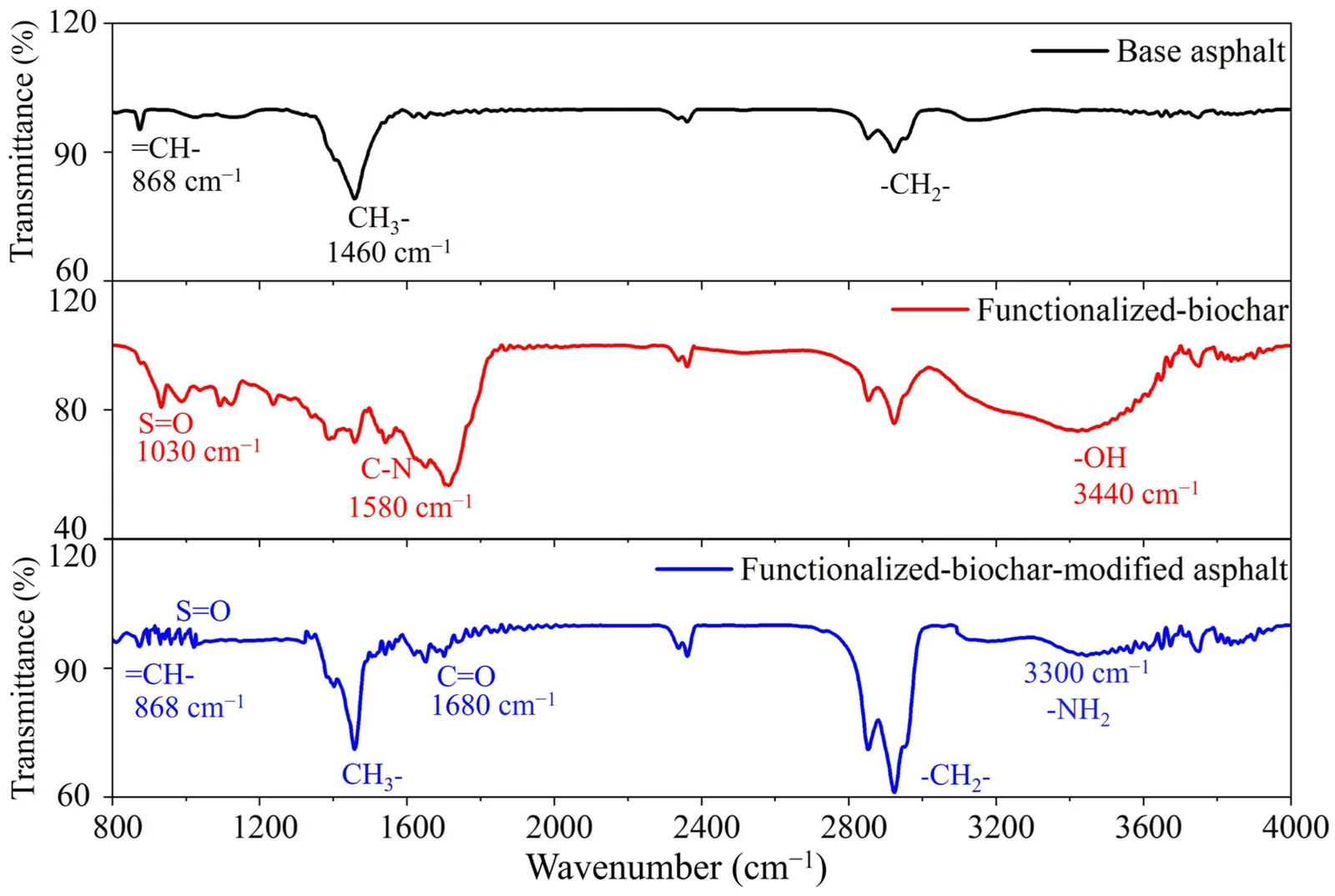
FTIR of base asphalt, functionalized-biochar, and functionalized-biochar-modified asphalt.

FTIR and FM were employed to investigate the interaction between functionalized-biochar and the base asphalt. As shown in Figure S4, the FTIR spectra of the base and functionalized-biochar-modified asphalt are highly similar, suggesting that the interaction is primarily governed by physical blending rather than a chemical reaction. No significant new peaks appear in the functionalized-biochar-modified asphalt, except for a distinct peak around 1600 cm^−1^, which corresponds to the intrinsic infrared characteristics of the functionalized-biochar (Figure 4).

**Figure 5:**
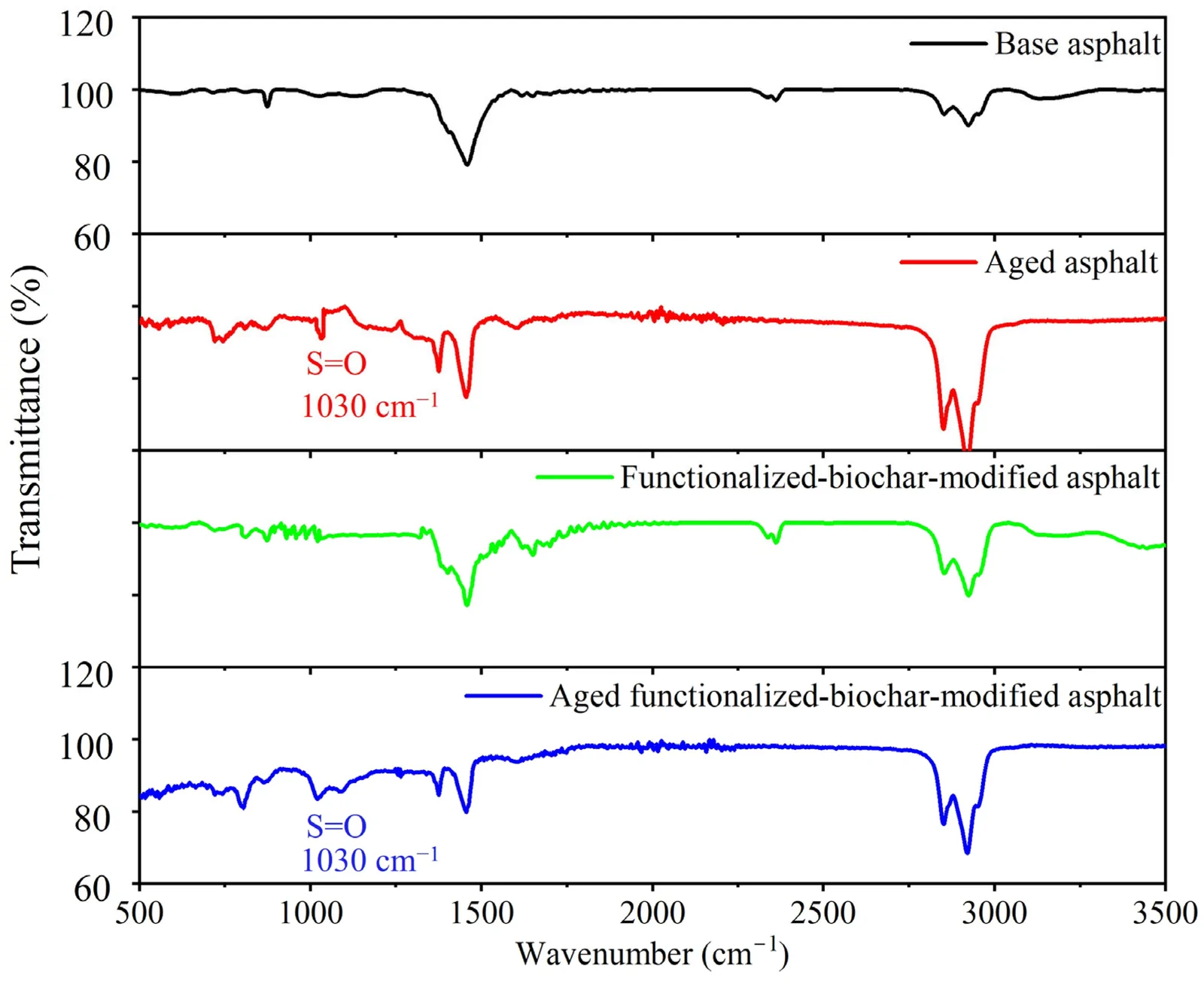
FTIR of base asphalt, aged asphalt, functionalized-biochar-modified asphalt, and aged functionalized-biochar-modified asphalt.

Figure 5 shows the FTIR spectra after RTFOT aging. A new absorption peak emerges at 1030 cm^−1^, characteristic of sulfoxide (S=O) formation, which correlates with the degree of asphalt oxidation. Although these peaks are relatively weak initially, they intensify with prolonged aging. Since the functionalized-biochar modification introduces C=O groups, carbonyl variations are not solely attributable to aging. The increased O-H peak (~3400 cm^−1^) post-aging likely reflects both asphalt oxidation and the inherent polar functional groups contributed by the biochar.

**Figure 6:**
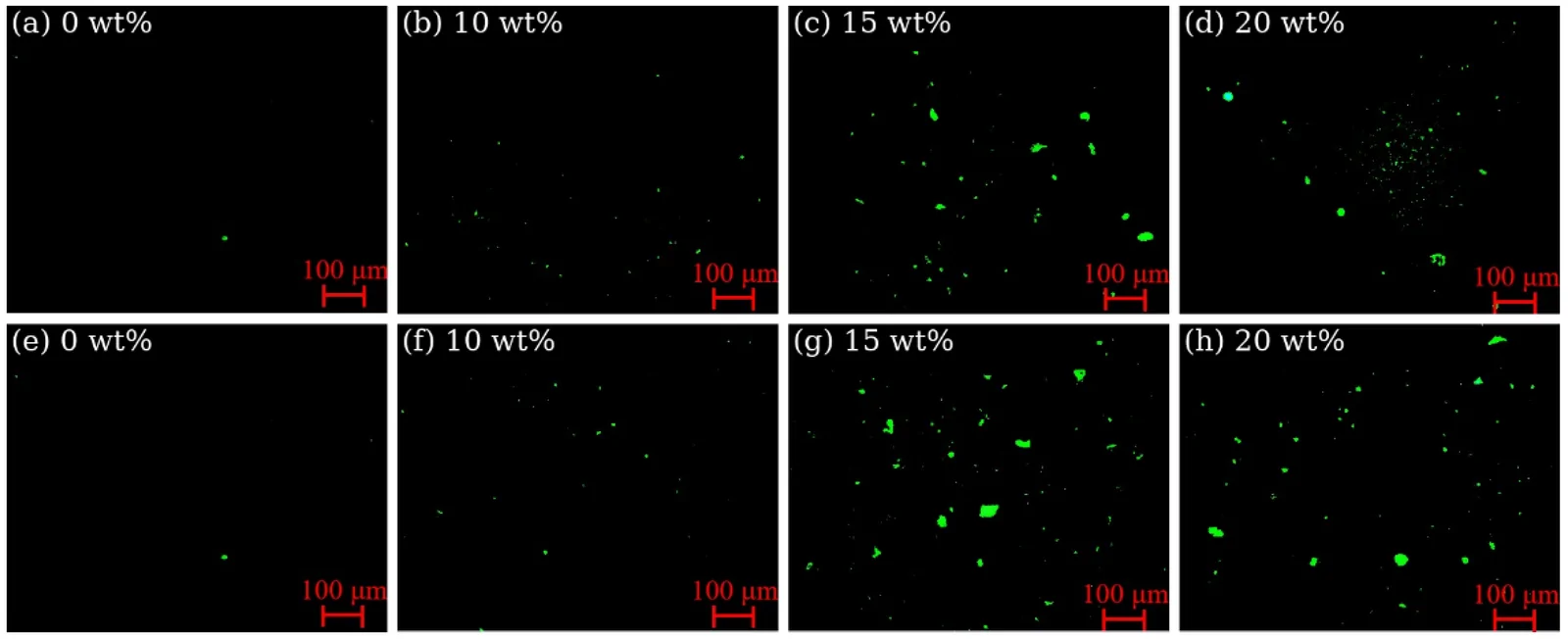
FM images of base asphalt and functionalized-biochar-modified asphalt with different dosages: (**a**–**d**) before aging, and (**e**–**h**) after aging.

FM analysis reveals the dispersion state of functionalized-biochar within the base asphalt. In contrast to the base asphalt, which exhibits negligible fluorescence, the functionalized-biochar-modified samples display fluorescence. As shown in Figure 6, the fluorescent domains were distributed throughout the observed fields. The sample containing 15 wt% functionalized-biochar exhibited a comparatively homogeneous spatial distribution, whereas more localized clustering was observed at 20 wt%. After RTFOT aging, the overall distribution pattern remained broadly similar. These observations suggest that 15 wt% functionalized-biochar provided a relatively favorable dispersion state under the present preparation conditions. The magnified images of the 15 wt% and 20 wt% samples before and after RTFOT aging are provided in Figure S5 in the Supporting Information.

The influence of biochar on the fracture resistance of asphalt mixtures is multi-faceted, heavily dependent on factors such as the feedstock type, dosage, dispersion quality, and the intrinsic properties of the base asphalt. While biochar’s three-dimensional network structure and inherent porosity typically enhance the fracture resistance [49,50], its susceptibility to agglomeration often undermines these benefits [51,52]. Consequently, precise optimization of dosage and surface modification is essential to balance reinforcing effects and suppress particle aggregation. The microscopic properties of asphalt are directly correlated with its macroscopic performance. Features such as functional group distribution and biochar network porosity dictate rheological behavior and durability through complex interfacial interactions, intermolecular forces, and physical crosslinking density. For instance, chemically functionalized-biochar improves microstructural stability, which is manifested as enhanced deformation resistance at the macroscopic level. Furthermore, microscopic fluctuations in carbonyl concentration influence molecular chain flexibility, thereby regulating the low-temperature brittleness threshold. Thus, fine-tuning these microscopic attributes allows for the targeted optimization of macroscopic road performance.

### 3.2. Physical Properties

#### 3.2.1. Conventional Properties Before Aging

**Figure 7:**
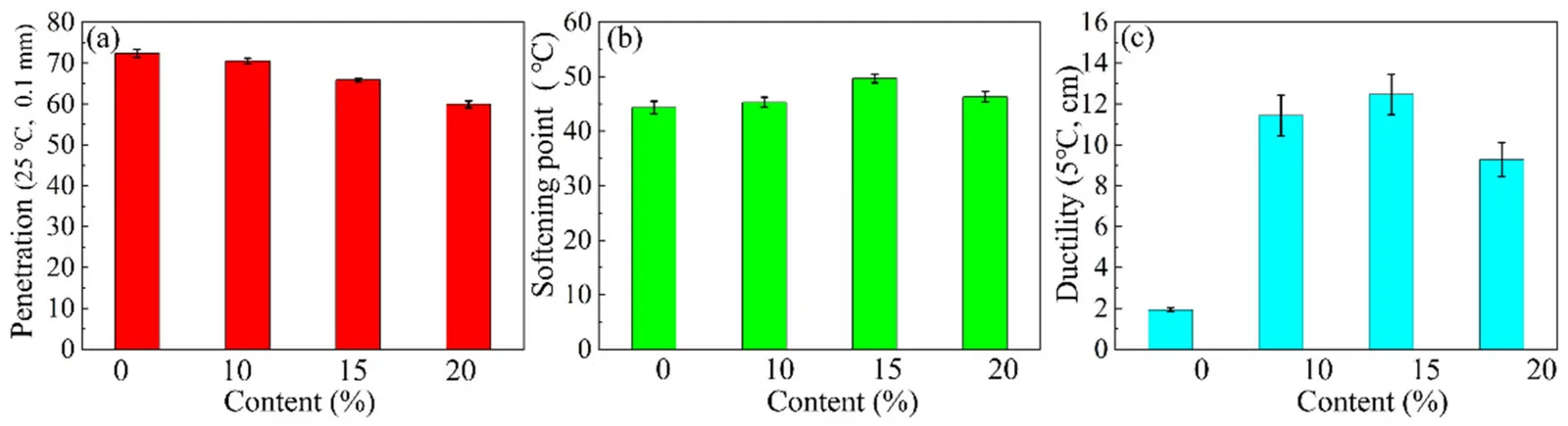
Physical properties of functionalized-biochar-modified asphalt: (**a**) penetration, (**b**) softening point, and (**c**) ductility.

The physical properties, including penetration, ductility, and softening point, were evaluated to assess the potential of functionalized-biochar as a modifier, and all tests were performed in triplicate to ensure data reliability. As shown in Figure 7, the modification primarily influences ductility. Post-modification, the penetration decreases, indicating increased consistency and hardness. This trend is consistent with previous observations for biochar-modified asphalts [53,54,55]. This reduction in penetration reflects typical particulate reinforcement behavior. Biochar particles, with specific surface areas in the range of 200–800 m^2^·g^−1^, provide extensive interfacial contact for molecular interactions [55]. Their large surface area enables strong adsorption of polar aromatics and light maltenes, effectively immobilizing part of the asphalt’s mobile phase and enhancing overall binder consistency. This mechanism is in line with the “skeleton structure and stiffening zone” concept proposed for biochar-modified binders [55]. The softening point initially increases with dosage and then decreases, consistently remaining higher than that of the base asphalt. The peak softening point at 15 wt% is attributed to the thickening effect of the solid biochar particles, which immobilize the mobile phase of asphalt through the adsorption of polar aromatics and light maltenes, thereby enhancing binder consistency. Similar increases in softening point with biochar incorporation have been reported [53,54], confirming the reinforcing effect of biochar on high-temperature performance. At 20 wt%, the softening point decline likely results from particle agglomeration reducing effective interfacial area, over-adsorption of polar aromatics depleting the lubricating fraction, and insufficient binder volume causing incomplete encapsulation. These effects collectively compromise the three-dimensional network, manifesting as a reduced softening point. The ductility follows a similar trend, peaking at 15 wt% (12.5 cm). In absolute terms, this represents an increase of 10.6 cm compared with the base asphalt (1.9 cm), corresponding to a relative improvement of 557.9%. At dosages of 10 wt% and 20 wt%, the absolute ductility values reached 11.4 cm and 9.3 cm, respectively (absolute increments of 9.5 cm and 7.4 cm; relative increases of 500% and 389%). It should be noted that the high percentage increases are partly attributable to the extremely low ductility of the 70# base asphalt at 5 °C (1.9 cm). Nevertheless, the absolute increment of 10.6 cm at 15 wt% indicates that the functionalized-biochar effectively improves the binder’s cohesive strength and flexibility without inducing excessive brittleness.

The selection of 15 wt% as the recommended dosage represents a balance between performance enhancement, and the onset of particle agglomeration. At 10 wt%, the biochar content is insufficient to fully develop the reinforcing network; at 15 wt%, favorable dispersion and interfacial interaction yield peak softening point (50.3 °C) and ductility (12.5 cm). At 20 wt%, both softening point and ductility decline, likely due to partial particle agglomeration that reduces effective surface area and disrupts the network structure. From a practical perspective, the incorporation of 15 wt% functionalized-biochar is compatible with conventional hot-mix asphalt production equipment, though the increased viscosity at this loading may require slightly higher mixing temperatures or extended mixing times. Fluorescence microscopy indicates satisfactory laboratory-scale storage stability without visible phase separation, though long-term segregation behavior warrants further investigation. While soybean powder served as a model precursor, the strategy is extendable to low-cost agricultural wastes to improve economic feasibility. The two-step functionalization uses industrially common reagents (HNO_3_ and HCHO/NaOH), but safety protocols and waste neutralization are necessary, and greener alternatives should be explored. Overall, pyrolysis reactors and high-shear mixers are already widely available, yet further validation of storage stability, economic feasibility, and environmental safety under real-world conditions remains essential prior to field deployment.

#### 3.2.2. Conventional Properties After Aging

To evaluate the aging resistance, the properties of the asphalt binders were assessed following the RTFOT protocol (Table 2). Figure S6a shows that mass loss during aging generally decreases and stabilizes with higher biochar content, suggesting a protective effect against volatile loss. However, given that mass loss reflects a balance between volatile evaporation and oxidative weight gain, a multi-indicator approach—including RPR (Residual Penetration Ratio) and SPI (Softening Point Increment)—was employed for a comprehensive assessment.

Post-aging, the base asphalt exhibited reduced penetration and an increased softening point, attributable to the volatilization of light components, enrichment of asphaltenes, and oxidative modification [56]. After aging, the asphalt becomes harder and more brittle, which restricts molecular mobility, reducing penetration and increasing softening point. The residual penetration ratio can be used to evaluate the aging resistance of asphalt, with a higher ratio indicating better aging resistance. As shown in Figure S6b,c, the modified asphalt consistently exhibits a higher RPR and a lower SPI compared to the base asphalt. Specifically, the functionalized-biochar-modified binder shows a maximum RPR increase of 3.15% and a reduced SPI of 0.7 °C relative to the control. These results signify that the functionalized-biochar effectively retards oxidative aging, consistent with previous findings [9,10,19].

Notably, while conventional physical property tests served only as initial screening for dosage optimization, the specific contributions of the modification treatment are further elucidated through DSR, aging indices, FTIR, and fluorescence microscopy. These characterizations distinguished the functionalized-biochar-modified asphalt from both the base and the biochar-modified samples, highlighting the superior rheological stability, anti-aging performance, and dispersion conferred by the optimized modification process.

### 3.3. High-Temperature Performance

**Figure 8:**
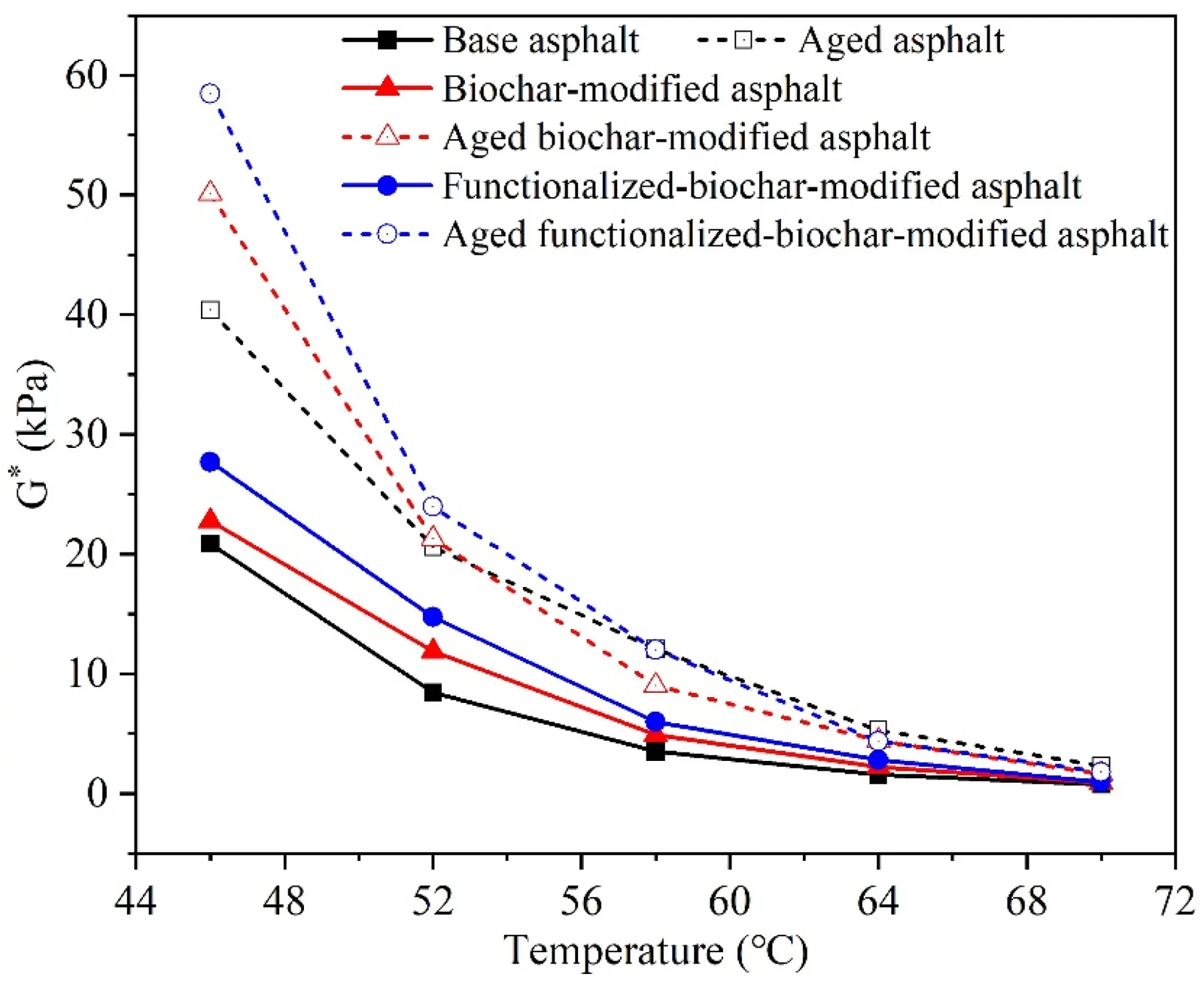
Temperature dependence of G* for base asphalt, modified asphalt, and aged asphalt.

Asphalt acts as a viscoelastic material whose performance is governed by temperature-dependent structural transitions. Key rheological parameters—G* (complex modulus), δ (phase angle), and G*/sin δ (rutting resistance factor)—were employed to characterize high-temperature deformation resistance. G* consists of elastic and viscous components, serving as a measure of the total resistance of asphalt during repeated shear deformation. It is defined as the ratio of maximum shear stress to maximum shear strain. As shown in Figure 8, the G* of all samples decreases rapidly with rising temperature before reaching a plateau. At 46 °C, the G* values for the functionalized-biochar-modified asphalt, biochar-modified asphalt, and base asphalt are 27.70, 22.76, and 20.86 kPa, respectively; these values converge to 0.95, 0.91, and 0.74 kPa at 70 °C. Across the entire temperature range, G* follows the order: functionalized-biochar-modified > biochar-modified > base asphalt. Following RTFOT aging, G* values increase across all samples, with maximum increments of 30.79, 27.37, and 19.53 kPa, respectively, underscoring the impact of short-term oxidative aging on stiffness.

**Figure 9:**
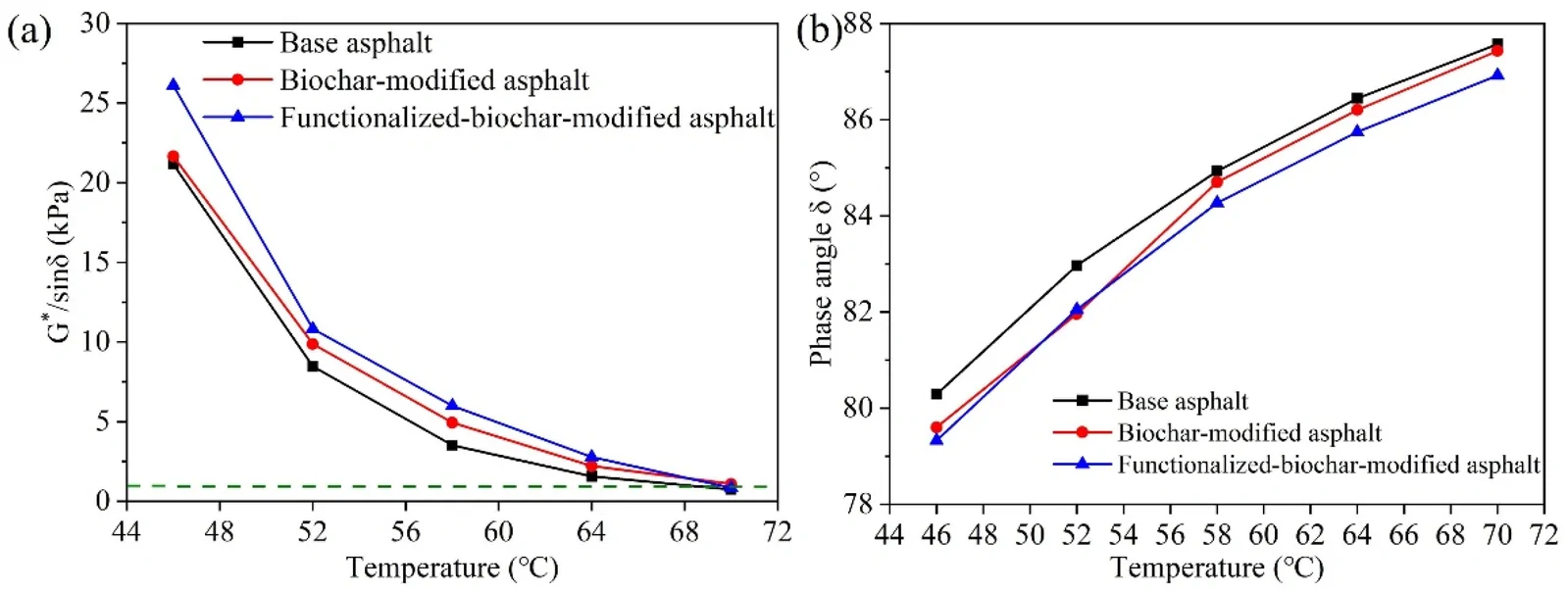
Temperature dependence of (**a**) G*/sin δ, and (**b**) δ of base asphalt and modified asphalt. The green dashed line represents the Superpave failure criterion (G*/sinδ = 1.0 kPa).

The rutting resistance factor G*/sin δ similarly demonstrated that both modified asphalts maintain linear viscoelastic behavior comparable to the base asphalt, as illustrated in Figure 9a. However, the modified samples consistently exhibit higher G*/sin δ values, indicating superior resistance to shear deformation at elevated temperatures. Specifically, at 46 °C, the functionalized-biochar-modified asphalt achieves a G*/sin δ of 26.10 kPa, compared to 21.66 kPa (biochar-modified asphalt) and 21.16 kPa (base asphalt). The failure temperature was elevated from 68.7 °C (base) to 69.8 °C (biochar) and 70.5 °C (functionalized-biochar), confirming the enhanced high-temperature thermal stability. It is important to note, however, that while the functionalized-biochar-modified binder consistently exhibits the highest G*/sin δ values, this modest increase in failure temperatures does not result in an upgrade of the high-temperature PG (Performance Grade) classification. The elevated G*/sin δ values instead indicate a greater margin of safety within the existing PG grade, which can be practically beneficial in terms of extended pavement service life under severe traffic and climatic conditions. The improvement is attributed to the “stiffening effect” of the biochar particles, which function as volume-filling reinforcements [55]. The large specific surface area and porous structure promote extensive interfacial adsorption of polar aromatics and light maltenes, effectively immobilizing the asphalt binder and increasing internal flow resistance. Furthermore, the functional groups introduced during modification facilitate stronger interfacial bonding, which reduces temperature sensitivity and enhances the material’s structural integrity. The elevation of softening point and G*/sin δ after incorporating functionalized-biochar conformed to the universal rule [57,58] that biochar additives strengthen asphalt high-temperature stiffness and anti-rutting capacity. Unlike the unmodified biochar used in the above studies, the two-step oxidation-hydroxymethyl treated soybean biochar, rich in intrinsic nitrogen polar groups, forms tighter interfacial bonding with asphalt components. Therefore, at a 15 wt% dosage, the increases in viscosity and rutting resistance observed in this work are more remarkable than those of biochar recorded in the previous literature [59]. It should be noted that G*/sin δ was evaluated only on unaged asphalt in this study, and testing after RTFOT aging was not performed. While the observed increases in G*/sin δ for the modified binders under unaged conditions indicate enhanced resistance to permanent deformation at elevated temperatures, the evaluation of rutting resistance after short-term aging, which more closely simulates field mixing and compaction conditions, remains an important aspect for future work. The absence of this analysis is acknowledged as a limitation of the present study.

As a viscoelastic material, asphalt resists flow deformation at high temperatures through its viscous components, enhancing rutting resistance, while its elastic components accommodate thermal shrinkage and relaxation at low temperatures, improving cracking resistance. The phase angle (δ) provides insight into the viscous–elastic balance. A larger δ indicates a higher proportion of viscous components and fewer elastic components, with δ = 0° representing purely elastic behavior and δ = 90° corresponding to a purely viscous fluid. As shown in Figure 9b, as temperature increases, δ rises, indicating a transition toward a more viscous-dominant state. Notably, the modified asphalts exhibit lower δ values compared to the base asphalt; at 46 °C, the functionalized-biochar-modified asphalt shows a 1.20% reduction in δ, suggesting a higher elastic component ratio. This increased elasticity is critical for mitigating permanent deformation and enhancing stress relaxation. As corroborated by FTIR (Figure S3), the successful grafting of polar groups (e.g., carbonyl and hydroxyl) onto the biochar surface enhances biochar–asphalt compatibility. This molecular-level interaction creates a more robust composite interface, providing a clear mechanistic basis for the superior rutting resistance and improved viscoelastic performance observed in the functionalized-biochar-modified system.

While the DSR-based G*/sin δ employed in this study is widely adopted for evaluating high-temperature performance of modified asphalt binders and suffices for comparative assessment of modifiers [57,58], we acknowledge that the experimental program does not encompass more comprehensive rheological characterization. Advanced methodologies—such as frequency sweep with master curve construction, MSCR (Multiple Stress Creep Recovery) for permanent deformation resistance, and LAS (Linear Amplitude Sweep) for fatigue performance—would provide complementary insights into the viscoelastic behavior of functionalized-biochar-modified asphalt. These techniques represent valuable directions for future work to fully elucidate the rheological benefits of nitrogen-rich, functionalized-biochar modifiers.

### 3.4. Anti-Aging Properties

**Figure 10:**
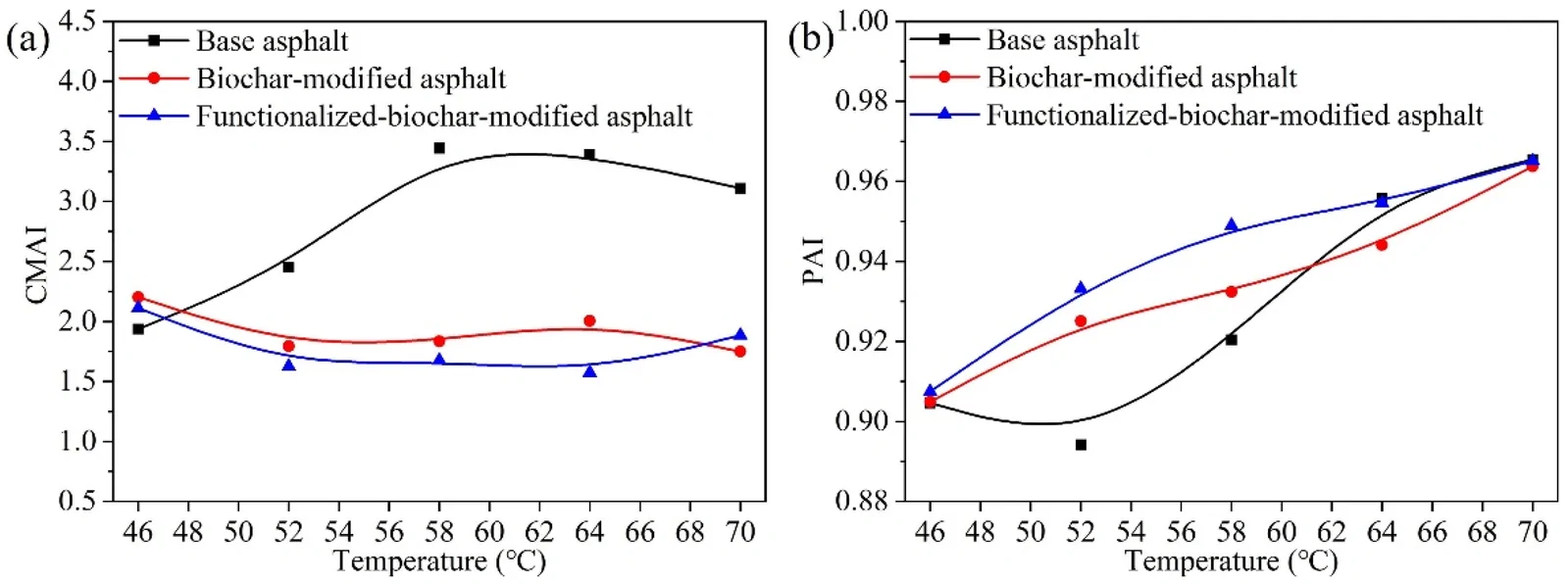
Asphalt aging indices: (**a**) complex modulus aging index, and (**b**) phase angle aging index.

To quantify the aging characteristics, rheological indices, CMAI (Complex Modulus Aging Index) and phase angle aging index (PAI), were derived to assess the durability of the modified asphalt, following established methodologies [12,60]. These indices are defined as [61,62,63]:

(3) $$\mathrm{CMAI}=G^{*}/G_{0}^{*}$$

(4) $$P\mathrm{AI}=\delta /\delta_{0}$$

where G* and $G_{0}^{*}$ (kPa) represent the complex modulus after and before aging, respectively, while δ and δ_0_ (°) are the phase angles after and before aging. A lower CMAI indicates less change in the complex modulus before and after aging, reflecting superior anti-aging properties. As shown in Figure 10a, all asphalt samples exhibit a CMAI > 1, with the base asphalt demonstrating the highest values, confirming its susceptibility to oxidative hardening. At 58 °C, the CMAI values for base asphalt, biochar-modified asphalt, and functionalized-biochar-modified asphalt are 3.44, 1.83 and 1.68, respectively. The lower CMAI of the functionalized-biochar-modified asphalt suggests that the surface functionalization strategy effectively mitigates oxidative stiffening.

This enhanced durability is attributed to a multi-scale stabilization mechanism. Chemically, the oxygen- and nitrogen-containing groups on the functionalized-biochar surface function as efficient radical scavengers, intercepting free radicals generated during thermo-oxidative aging [13,64]. The delocalized π-electron system within the biochar skeleton further promotes the quenching of carbon-centered radicals, thereby retarding the formation of carbonyl and sulfoxide species. Physically, the porous morphology of biochar acts as a tortuous barrier that reduces oxygen permeability by an estimated 25–40% [59,65]. The smaller biochar particles act as more efficient rigid fillers, reinforcing this inhibitory effect. Two primary physical mechanisms contribute to this behavior: (i) greater volumetric filling reduces the accessibility of asphalt components prone to oxidation, and (ii) the complex fibrous structure of biochar promotes stronger adhesion with the base asphalt, physically limiting molecular-level oxidative reactions. Furthermore, the adsorption of light maltenes onto the high-surface-area biochar matrix effectively immobilizes volatile components, suppressing their evaporative loss. This synergy, combining reactive scavenging and physical barrier effects, lowers the oxidation rate of the binder matrix. The decreasing CMAI observed aligns with existing research [53,57,59] which reported that porous biochar delays asphalt oxidation by physical UV shielding and oxygen isolation. However, their unmodified biochar lacks inherent nitrogen species and grafted polar groups, so anti-aging performance may be limited to simple physical protection. In comparison, nitrogen-rich soybean biochar modified via two-step oxidation–hydroxymethylation may realize dual anti-aging effects: the porous structure blocks oxygen diffusion, while intrinsic pyridinic-N and pyrrolic-N species scavenge oxidative free radicals [66,67].

The PAI provides complementary insights into the evolution of viscoelastic behavior (Figure 10b). A PAI closer to 1.0 indicates higher stability in the viscoelastic balance post-aging. The functionalized-biochar-modified asphalt consistently exhibits the highest PAI values (e.g., 0.97 at 70 °C), compared to 0.89 for the base asphalt (at 52 °C), confirming that the modification preserves the material’s elastic–viscous equilibrium. As temperature rises, the gradual increase in PAI across all samples reflects a natural shift toward a more viscous-dominant state, yet the modified system maintains superior resistance to aging-induced embrittlement. In summary, the functionalized-biochar acts as a multi-functional additive: its surface chemistry actively inhibits radical-mediated oxidative pathways, while its structural porosity imposes physical constraints on oxygen diffusion and volatile release. This integrated mechanism fundamentally bolsters the long-term aging resistance of the asphalt binder.

### 3.5. Implication of Using Soybean-Derived Biochar in Asphalt

Soybean-derived, nitrogen-rich biochar emerges as an ideal candidate due to its high specific surface area and tunable surface chemistry. Its nitrogen-containing functional groups are considered to play a key role in governing adsorption performance through several potential synergistic mechanisms [26,27]. Firstly, the coexistence of electron-rich (e.g., amine, hydroxyl) and electron-deficient (e.g., amide, carboxyl) domains may facilitate the capture of molecules via varying electronic interactions. Secondly, the high electronegativity of nitrogen atoms could foster robust hydrogen bonding with electronegative oxygen/sulfur species within the asphalt. Finally, the aromatic polycyclic framework of biochar is reported to enable strong π–π stacking and CH_2_–π interactions [67] with aliphatic asphalt chains. Collectively, these intermolecular forces, including electrostatic attraction, dipole–dipole interactions, and hydrogen bonding [26,27] are hypothesized to contribute not only to hazardous gases sequestration but also to reinforcement of the asphalt–biochar interface, potentially retarding oxidative hardening and offering potential environmental benefits aligned with carbon neutrality goals in infrastructure development. It should be noted that the proposed interaction mechanisms discussed above are reasonable inferences drawn from the literature on related carbonaceous systems [26,27,67], rather than direct conclusions derived from the experimental program of this study. Direct experimental verification of these specific mechanisms remains an important direction for future work. Furthermore, it is important to emphasize that hazardous gases emissions, carbon balance, and life-cycle impacts were not directly evaluated in the present study. Therefore, the environmental benefits should be viewed as potential advantages or literature-based theoretical projections, rather than experimentally demonstrated outcomes. Direct hazardous gases emission testing and comprehensive life-cycle assessment are therefore recommended as important directions for future work to substantiate these potential advantages.

## 4. Conclusions

This study investigates the performance enhancement of asphalt binders using functionalized, soybean-derived biochar. Key findings are as follows:

(1) Tailored Modification: Biochar was produced via pyrolysis at 300 °C. A two-step oxidation-hydroxymethylation process successfully functionalized soybean-derived biochar, enhancing its interfacial compatibility with the base asphalt.
(2) Macroscopic Property Enhancement: At the recommended dosage of 15 wt%, the functionalized-biochar bolstered high-temperature stability. The binder exhibited a 6 °C increase in softening point, an absolute increase in ductility from 1.9 cm to 12.5 cm (a 10.6 cm increment, corresponding to a relative improvement of 557.9%), and a 17% reduction in penetration, indicating superior mechanical resilience and reduced temperature sensitivity under the laboratory conditions tested.
(3) Rheological and Aging Resistance: The functionalized-biochar-modified asphalt exhibited superior rheological stability, evidenced by lower δ and higher G*/sin δ within the same performance grade. The CMAI was reduced to 1.68, compared to 3.44 for the base asphalt, and the PAI was effectively stabilized, confirming the material’s enhanced oxidative durability after short-term aging protocols.

The functionalized-biochar-modified binder is suitable for heavy traffic pavements, high-temperature regions, and environmentally sensitive areas requiring hazardous gases mitigation. Based on comprehensive evaluation, 15 wt% is recommended as the optimal dosage, balancing performance enhancement with the onset of particle agglomeration. This dosage is compatible with conventional HMA production, though slightly higher mixing temperatures may be required. The enhanced aging and rutting resistance suggest reduced oxidative hardening and potentially lower life-cycle costs, while the biochar’s porous structure offers additional environmental benefits. However, challenges remain; scaling up the chemical modification require optimization of reagent recovery and cost control; long-term storage stability was not assessed; and the optimal dosage may vary with base asphalt source and climatic conditions, warranting small-scale mixture trials prior to field deployment.

Prospects: The present study demonstrates improved high-temperature rheological properties and aging-related parameters for the functionalized-biochar-modified asphalt. However, several limitations should be acknowledged: MSCR, low-temperature cracking parameters, and G*/sin δ after RTFOT aging were not evaluated. Future work will therefore focus on (i) long-term durability using PAV protocols; (ii) low-temperature flexibility and storage stability; (iii) MSCR and Glover–Rowe analyses for comprehensive rheological characterization; and (iv) composite modification strategies to achieve balanced performance over a wider temperature range.

## Figures and Tables

**Table 1:** Physical properties of base asphalt.

Parameter	Unit	Test Data	Specification
Penetration (25 °C)	0.1 mm	72.4	60–80
Softening point	°C	44.3	44–54
Ductility (5 °C)	cm	1.9	-
Density (15 °C)	g/cm^3^	1.04	-
Solubility (trichlorethylene)	%	99.84	≥99.5

**Table 2:** Technical properties of modified asphalt after RTFOT.

Type of Asphalt	Content (%)	Mass Loss (%)	RPR (%)	SPI (°C)
Base asphalt	0	−0.232	69.76	7.6
Modified asphalt	10	−0.211	71.07	7.3
	15	−0.213	72.91	6.9
	20	−0.209	72.64	7.1

## Data Availability

The original contributions presented in this study are included in the article/Supplementary Materials. Further inquiries can be directed to the corresponding author.

## Supplementary Materials

The following supporting information can be downloaded at: https://www.mdpi.com/article/10.3390/ma19173639/s1, Figure S1: (a) Pore size distribution, (b) N_2_ adsorption-desorption isotherms; Figure S2: SEM images of biochar at different temperatures, (a) 200 °C, (b) 300 °C, (c) 400 °C, (d) 500 °C; Figure S3: Fourier transform infrared (FTIR) spectra of raw biochar and functionalized biochar; Figure S4: The FTIR of base asphalt and functionalized biochar-modified asphalt with different dosages; Figure S5: Enlarged fluorescence microscopy images comparing the spatial distribution of fluorescent domains in asphalt samples containing 15 and 20 wt% functionalized-biochar before and after RTFOT aging. The enlarged views are derived from the corresponding fields shown in Figure 6; Figure S6: Properties of aged asphalt modified with functionalizedbiochar at different dosages, (a) mass loss, (b) residual penetration ratio, (c) softening point increment; Table S1: Elemental analysis of biochar at different temperatures.
