# Supplementary material for: Research on the Rutting Resistance of Asphalt Improved by Nitrogen-Rich Soybean Biochar
Source: Materials (Basel). 2026 Aug 27;19(17):3639. doi: 10.3390/ma19173639 (PMC13566927; doi:10.3390/ma19173639)
Supplement: Supplementary file 1 [file materials-19-03639-s001.zip › materials-4467031-supplementary.pdf]

Research on the Rutting Resistance of Asphalt Improved by Nitrogen-Rich Soybean Biochar

Cuicui Sun [1], Zhe Li [1], Junxia Yang [2], Xuanchen Zhou [1], Yanling Wu [1], Haocheng Zhang [1], Changhao Si [1], Xiaofeng Tian [1,*], Chiara Riccardi [3] and Dedong Guo [1]

[1] Shandong Key Laboratory of Technologies and Systems for Intelligent Construction Equipment, School of Civil Engineering, Shandong Jiaotong University, Jinan 250300, China
[2] Shandong Science and Technology Service Development and Promotion Center, Jinan 250300, China
[3] Department of Civil and Industrial Engineering, University of Pisa, Largo Lucio Lazzarino 1, 56126 Pisa, Italy
* Correspondence: tianxiaofeng@sdjtu.edu.cn

Preparation and physical performance of biochar

1. Determination method of biochar oil absorption value D

A sufficient volume of biochar samples was dried in a 125°C drying box for 1 h and then cooled to room temperature in a dryer. First, dibutyl phthalate (DBP) is added to the reservoir of the microtiter. Second, taking about 0.2 g dry biochar (m) on the glass plate, using micro titer tube DBP drops on the biochar sample with drop acceleration of 2-4 drops per second. At the same time, continuously uniform rolling by the glass bar, until the biochar all oil infiltration evenly. Then, add DBP at slow speed with glass rod constantly stirring and rolling. Finally, the biochar is completely rolled on the glass bar, and leaving no oil trace on the glass plate is the end point. At this time, the DBP consumed in the titer tube is read, which is denoted as V, in ml. The oil absorption value D is as follows.

$$D = \frac{V}{m} \times 100$$

2. The determination method of biochar iodine absorption value I

Adequate amount biochar samples were placed on a petri dish in 125°C for 1 h and placed in an oven to cool to room temperature. First, 0.1 g of biochar (expressed as m) and 5 ml of iodine standard solution (expressed as V_0^*) were placed in a centrifugal tube, which was vibrated for 1 minute with the ultrasonic oscillator and then centrifuged at 6000 rpm for 3 min. Then, 4 ml of the supernatant (as indicated V_1^*) was placed into the beaker and titrated with the standard solution of sodium thiosulfate (concentration C) until the solution becomes pale yellow. The inner wall of the glass was washed with distilled water, then a drop of starch solution was added to the glass. And the sodium thiosulfate standard solution was dropped into the glass until it became colorless, and the reading of the drip tube was recorded as V_1. Moreover, the same method requires adding a set of samples without biochar, in which the standard solution of sodium thiosulfate is V_0. The iodine absorption value I is as follows.

$$I = \frac{V_0 - V_1}{V_1^*} \times V_0^* \times C \times \frac{126.91}{m}$$

3. The determination method of the total acidity of T

0.05 g of unmodified raw biochar and functionalized biochar were placed into a numbered 100 ml conical flask. Adding 10 ml of 0.25 mol/L of sodium hydroxide solution and 20 ml of distilled

water to the conical flask and sonicate at a constant temperature of 50°C. After the solution is cooled, adding 3 drops of methyl red indicator to the conical flask. The reverse titration was performed with 0.23 mol/L of dilute hydrochloric acid, until the solution changed from yellow to red. Tittration volumes V and V_0 of functionalized biochar and raw biochar were recorded. The total acidity of T is as follows.

$$T=\frac{(V_0-V)\times C_{HCl}}{m}$$

4. Determination of the carboxyl and lactone groups content P

0.05 g of raw biochar and functionalized biochar were placed into a 100 ml conical flask. Adding 10 ml of 0.25 mol /L of sodium carbonate solution and 20 ml of distilled water to the conical flask and sonicated at 50°C for 30 min. After the solution was cooled, 15 ml of 0.23 mol/L of hydrochloric acid was added to the conical flask and was sonicated again at 50°C for 30 min. After cooling, adding 3 drops of ethanol dissolved phenolphthalein indicator into the conical flask. After dispersion evenly, reverse titration with 0.25 mol/L of sodium hydroxide until the solution changes from colorless to red. The consumed NaOH alkaline solution volumes V^* and V_0^* of the functionalized biochar and raw biochar were recorded. The carboxyl and lactone groups P is as follows.

$$P=\frac{(V_0^*-V^*)\times C_{NaOH}}{m}$$

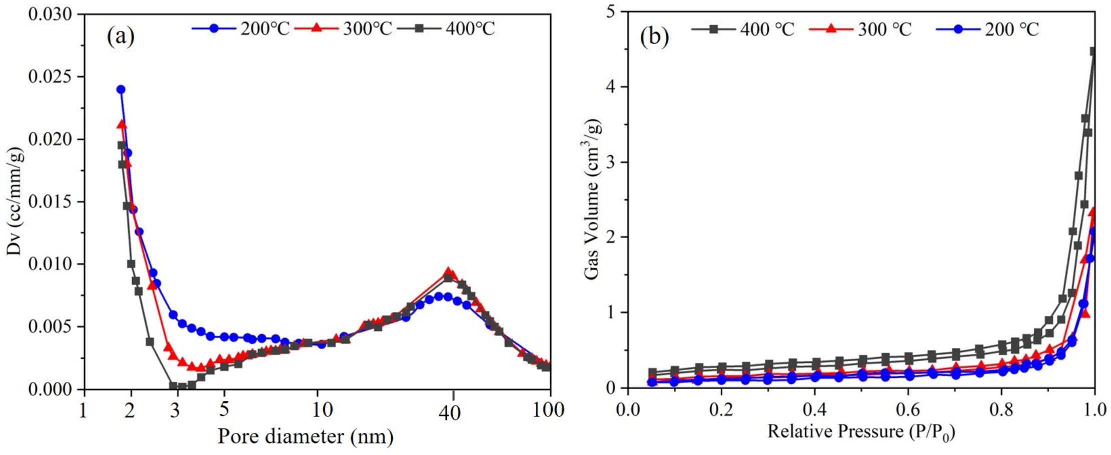

Figure S1. (a) Pore size distribution, (b) N_2 adsorption-desorption isotherms

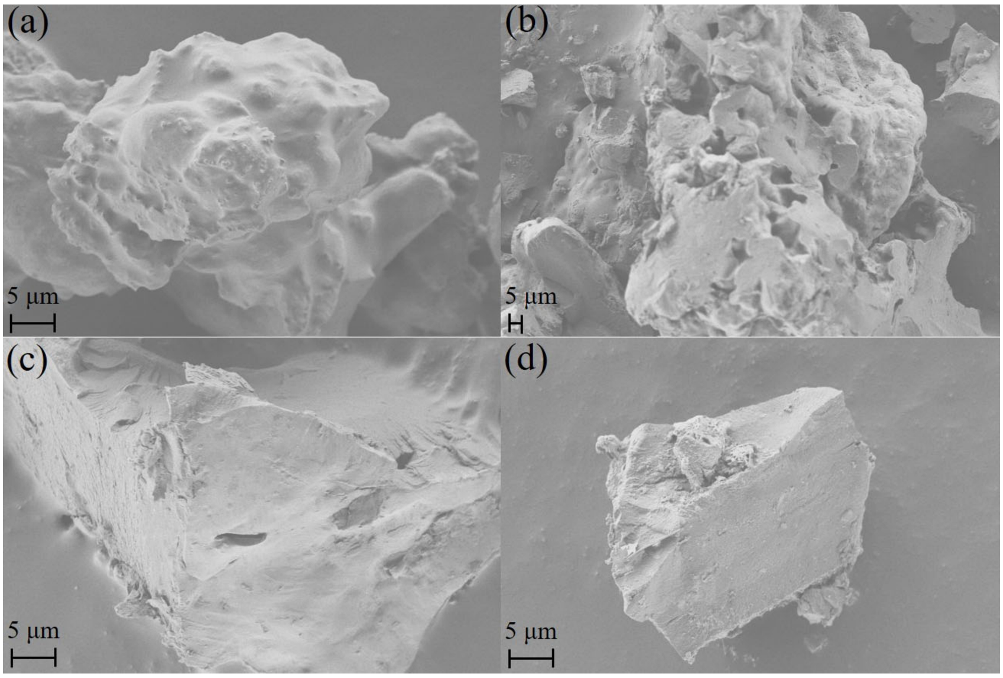

Figure S2. SEM images of biochar at different temperatures, (a) 200 °C, (b) 300 °C, (c) 400 °C, (d) 500 °C

Table S1. Elemental analysis of biochar at different temperatures.

Element, wt.%	200 °C	300 °C	400 °C	500 °C
Carbon	72.36	69.45	48.36	48.33
Oxygen	17.01	16.75	26.92	46.47
Nitrogen	6.83	7.87	2.67	2.46
Potassium	2.5	5.59	37.73	29.33
hydrogen	5.08	4.23	3.25	2.54
Fixed-C	44.6	52.8	63.3	65.8

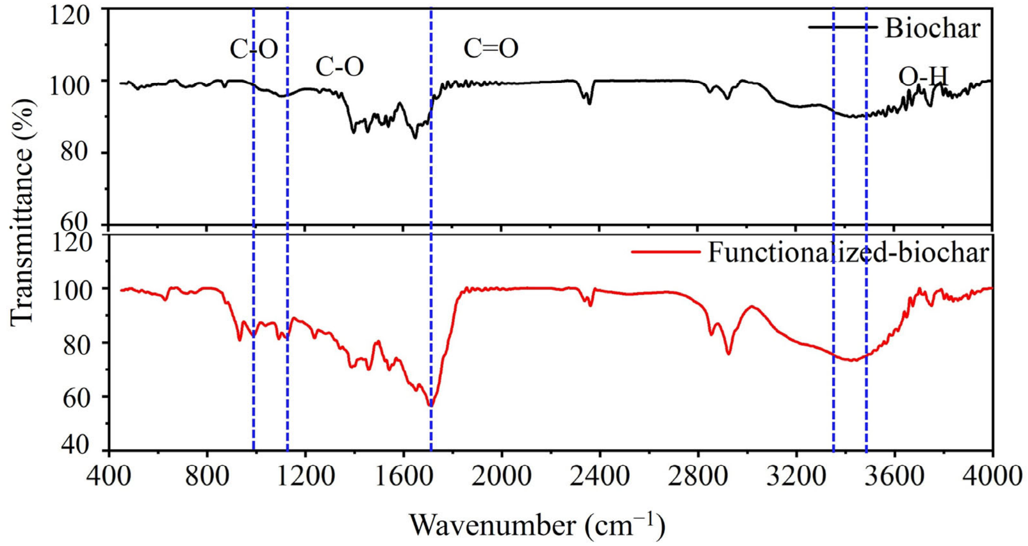

Figure S3. Fourier transform infrared (FTIR) spectra of raw biochar and functionalized biochar.

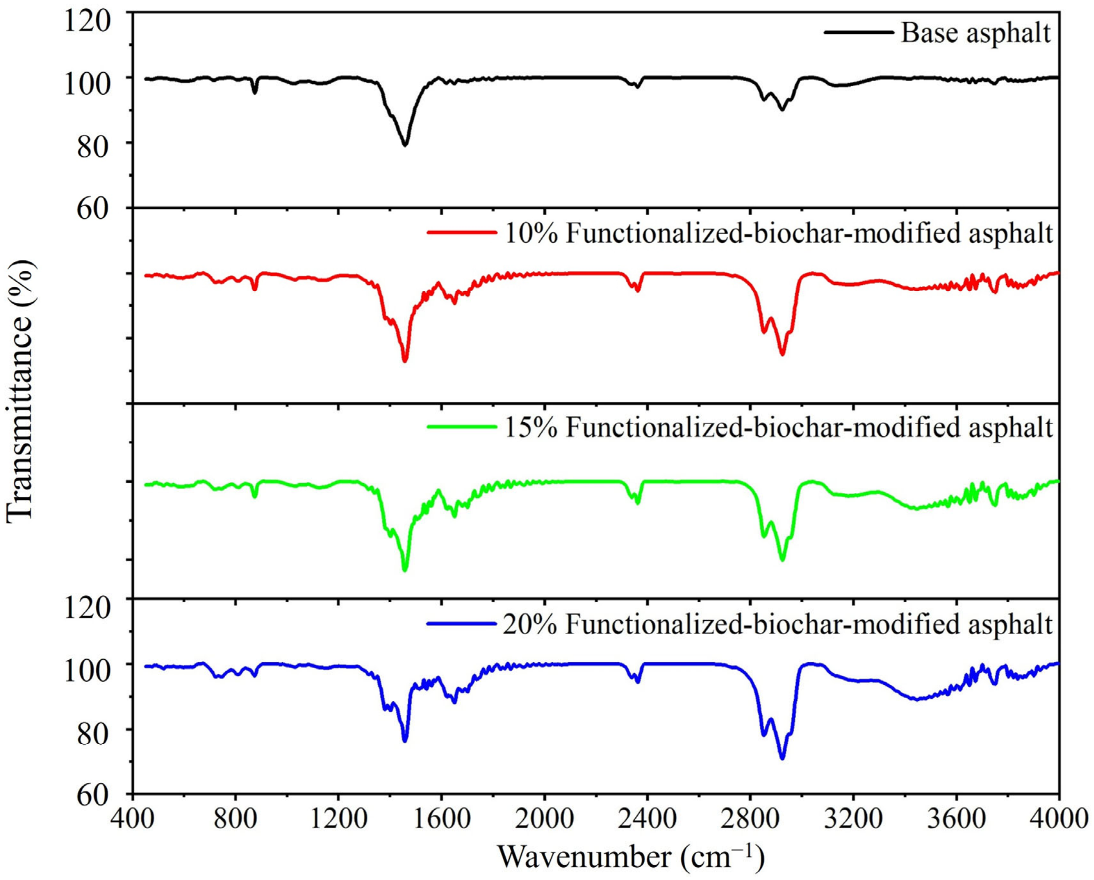

Figure S4. The FTIR of base asphalt and functionalized biochar-modified asphalt with different dosages.

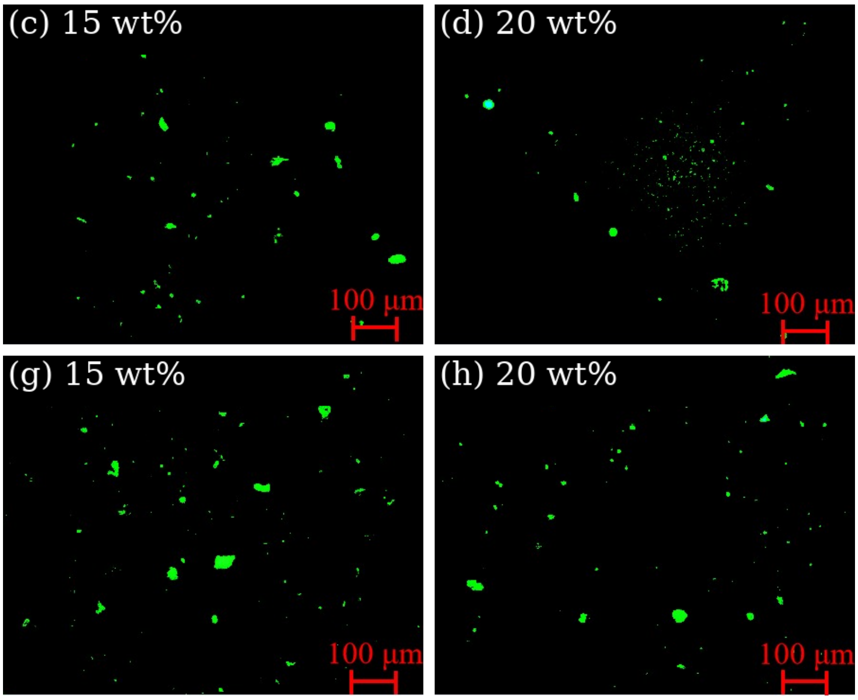

Figure S5. Enlarged fluorescence microscopy images comparing the spatial distribution of fluorescent domains in asphalt samples containing 15 and 20 wt% functionalized-biochar before and after RTFOT aging. The enlarged views are derived from the corresponding fields shown in Figure 6.

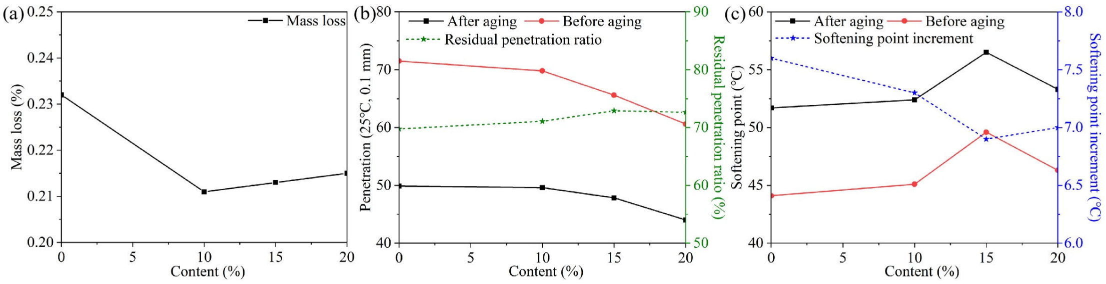

Figure S6. Properties of aged asphalt modified with functionalizedbiochar at different dosages, (a) mass loss, (b) residual penetration ratio, (c) softening point increment.
